# [1,2-Bis(diphenyl­phosphan­yl)ethane-κ^2^ *P*,*P*′]dichloridopalladium(II) dimethyl sulfoxide monosolvate

**DOI:** 10.1107/S1600536812028310

**Published:** 2012-06-30

**Authors:** Ismail Warad, Abdullah S. Aldwayyan, Fahad M. Al-Jekhedab, M. Iqbal Choudhary, Sammer Yousuf

**Affiliations:** a Department of Chemistry, College of Science, King Saud University, PO Box 2455, Riyadh 11451, Saudi Arabia; b Department of Physics and Astronomy, College of Science, King Saud University, PO Box 2455, Riyadh 11451, Saudi Arabia; c Electronics, Communications and Photonics Program, King Abdulaziz City for Science and Technology, PO Box 6086, Riyadh 11442, Saudi Arabia; d H.E.J. Research Institute of Chemistry, International Center for Chemical and Biological Sciences, University of Karachi, Karachi 75270, Pakistan

## Abstract

In the title compound, [PdCl_2_(C_26_H_24_P_2_)]·C_2_H_6_OS, the Pd^II^ atom adopts a distorted *cis*-PdCl_2_P_2_ square-planar coordination geometry. The five-membered chelate ring adopts an envelope conformation with a methyl­ene C atom in the flap position. The S and C atoms of the dimethyl sulfoxide (DMSO) solvent mol­ecule are disordered over two sets of sites in a 0.8976 (18):0.1024 (18) ratio. The DMSO O atom accepts three C—H⋯O hydrogen bonds from an adjacent complex mol­ecule.

## Related literature

For the previous reports of crystal structures of this metal complex (unsolvated or with other solvents), see: Xu *et al.* (2008 ▶); Batsanov *et al.* (2001 ▶); Steffen & Palenik (1976 ▶); Singh *et al.* (1995 ▶).
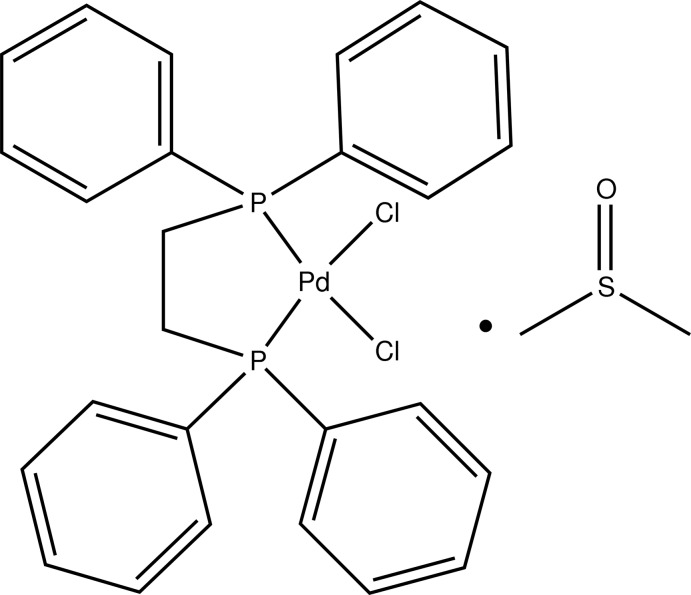


## Experimental

### 

#### Crystal data

[PdCl_2_(C_26_H_24_P_2_)]·C_2_H_6_OS
*M* *_r_* = 653.82
Triclinic, 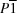

*a* = 8.4091 (3) Å
*b* = 11.4745 (4) Å
*c* = 16.8098 (6) Å
α = 73.674 (1)°
β = 79.066 (1)°
γ = 68.634 (1)°
*V* = 1442.67 (9) Å^3^
*Z* = 2
Mo *K*α radiation
μ = 1.03 mm^−1^
*T* = 293 K
0.30 × 0.23 × 0.11 mm

#### Data collection

Bruker SMART APEX CCD diffractometer
Absorption correction: multi-scan (*SADABS*; Bruker, 2000 ▶) *T*_min_ = 0.747, *T*_max_ = 0.895
19016 measured reflections
6610 independent reflections
5863 reflections with *I* > 2σ(*I*)
*R*_int_ = 0.022

#### Refinement

*R*[*F* ^2^ > 2σ(*F* ^2^)] = 0.027
*wR*(*F* ^2^) = 0.069
*S* = 1.03
6610 reflections
326 parameters
3 restraints
H-atom parameters constrained
Δρ_max_ = 0.50 e Å^−3^
Δρ_min_ = −0.32 e Å^−3^

### 

Data collection: *SMART* (Bruker, 2000 ▶); cell refinement: *SAINT* (Bruker, 2000 ▶); data reduction: *SAINT*; program(s) used to solve structure: *SHELXS97* (Sheldrick, 2008 ▶); program(s) used to refine structure: *SHELXL97* (Sheldrick, 2008 ▶); molecular graphics: *SHELXTL* (Sheldrick, 2008 ▶); software used to prepare material for publication: *SHELXTL*, *PARST* (Nardelli, 1995 ▶) and *PLATON* (Spek, 2009 ▶).

## Supplementary Material

Crystal structure: contains datablock(s) global, I. DOI: 10.1107/S1600536812028310/hb6864sup1.cif

Structure factors: contains datablock(s) I. DOI: 10.1107/S1600536812028310/hb6864Isup2.hkl

Additional supplementary materials:  crystallographic information; 3D view; checkCIF report

## Figures and Tables

**Table 1:** Selected bond lengths (Å)

Pd1—P2	2.2336 (5)
Pd1—P1	2.2355 (5)
Pd1—Cl1	2.3481 (6)
Pd1—Cl2	2.3613 (5)

**Table 2:** Hydrogen-bond geometry (Å, °)

*D*—H⋯*A*	*D*—H	H⋯*A*	*D*⋯*A*	*D*—H⋯*A*
C2—H2⋯O1	0.93	2.49	3.348 (4)	153
C20—H20⋯O1	0.93	2.59	3.495 (4)	165
C26—H26*A*⋯O1	0.97	2.44	3.410 (3)	174

**Fig. 1.**
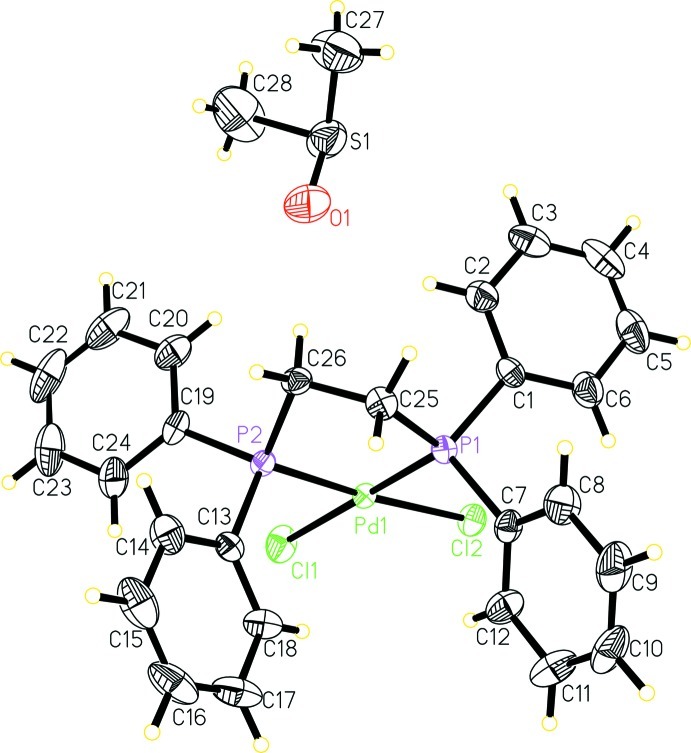
The molecular structure of (I) with displacement ellipsoids drawn at 30% probability level.

**Fig. 2.**
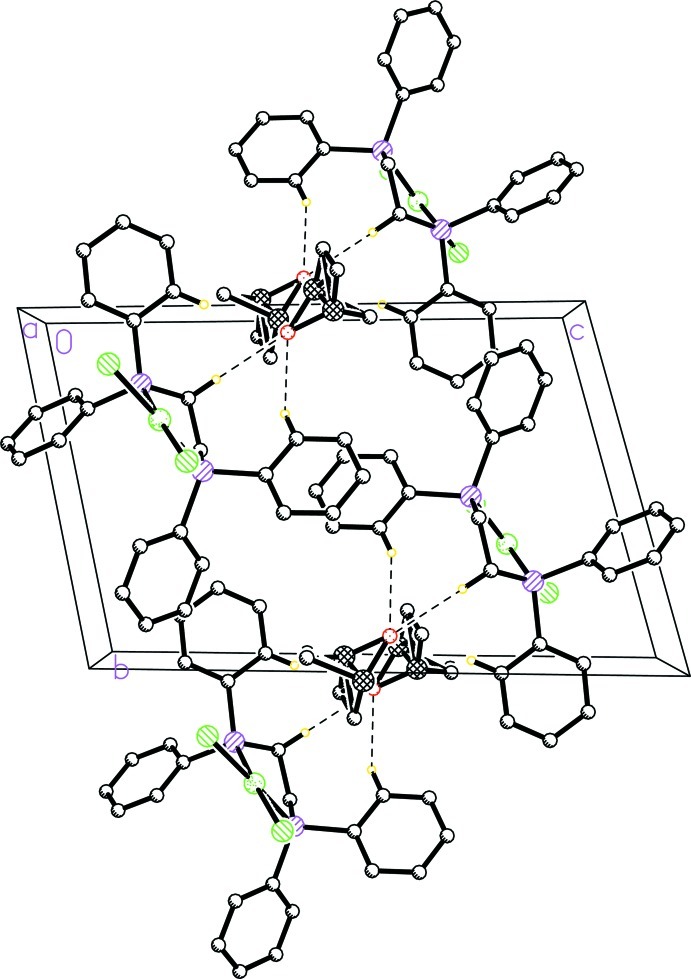
The crystal packing of the title compound I. Only hydrogen atoms involved in hydrogen bonding are shown.

## supplementary crystallographic information

### Comment

Metal complexes containing chelating diphosphines as ligands have been employed in numerous catalytic processes. A major advantage of these ligands is variation in their potential catalytic reactivity by varying the molecular properties of the phosphine. The title compound was synthesized as a part of our ongoing research to study the properties of metal complexes with chelating ligands.

The crystal structure of title compound, [PdCl_2_C_26_H_24_P_2_)].C_6_H_6_SO, consists of a bidentate diphenyl phosphine ligand and two chloride atoms coordinated with Pd(II) to adapt a distorted square planner geometry, along with an independent molecule of dimethyl sulfoxide (DMSO) as solvent (Fig. 1). The five membered metallocycle (Pd1/P1/P2/C25—C26) adopts an envelop conformation [Q= 0.466 (19) Å and φ = 301.14 (17)°] with maximum deviation of 0.318 (2) Å for C26 atom from the least square plane. The Structural report of the compound is similar to many previously published reports with the difference that it has a DMSO solvate (Xu *et al.* 2008, Batsanov *et al.* 2001,Steffen & Palenik, 1976, Singh *et al.* 1995). The coordination environment around the Pd(II) ion is such that the two phosphorous atoms of the bidentate diphenyl phosphine ligand [Pd1–P1 = 2.2355 (5) Å, Pd1–P2 = 2.2336 (5) Å] and two chloride atoms [Pd1–Cl1= 2.3481 (6) Å, Pd1–Cl2= 2.3613 (5) Å] are assembled at four corners of square to adapt square pyramidal arrangement. The S1, C28 and C29 atoms of DMSO molecule are disordered at two positions,with relative occupancies of 0.89 (18):0.10 (18) for the isotropically refined major (S1/C27/C28) and minor (S1'/C27'/C28') components, respectively. All bond lengths are in agreement with the previously reported crystal structures (Xu *et al.* 2008, Batsanov *et al.* 2001, Steffen & Palenik, 1976, Singh *et al.* 1995). The oxygen atom of DMSO playing an important role in the crystal structure by forming C2—H2···O1, C20—H20···O1 and C26—H26A···O1 hydrogen bonds (Table 2, Fig. 2).

The compound was evaluated for its b-glucoronidase inhibiton activity against D-saccharic acid as standard (IC_50_ 45.75 ± 2.16 mM) and found as weak inhibitor (IC_50_ 197.3 ± 12.2 mM)

### Experimental

Equimolar amounts of dichlorobis(acetonitrile) palladium(II) (0.10 g, 0.26 mmol) was dissolved in 10 ml dry dichloromethane and mixed with equivalent amount of 1,2-ethanediylbis(diphenylphosphine) (0.11 g, 0.27 mmol). The resultant mixture was stirred under inert atmosphere (Ar) for about one hour. The solution was concentrated under reduced pressure to 1 ml volume. The product was precipitated by the addition of 30 ml of hexane. Then filtered off, washed with 40 ml of diethyl ether and dried under vacuum to obtained 0.12 g of title compound I (yield 78%). 20 mg of the product was dissolved in 5 ml of dry DMSO for crystallization. After one week, light orange color crystals were obtained which were found to be suitable for X-ray diffraction data collection. All chemicals were purchased from Acros (Belgium).

### Refinement

H Atoms on methyl, methylene and methine were positioned geometrically with C—H = 0.96 Å, 0.97 Å and 0.93 Å respectively, and constrained to ride on their parent atoms with *U*_iso_(H)= 1.2*U*_eq_ (CH_2_, CH) and 1.5*U*_eq_(CH_3_).

### Crystal data

**Table:**

[PdCl_2_(C_26_H_24_P_2_)]·C_2_H_6_OS	*Z* = 2
*M**_r_* = 653.82	*F*(000) = 664
Triclinic, *P*1	*D*_x_ = 1.505 Mg m^−^^3^
*a* = 8.4091 (3) Å	Mo *K*α radiation, λ = 0.71073 Å
*b* = 11.4745 (4) Å	Cell parameters from 8463 reflections
*c* = 16.8098 (6) Å	θ = 2.5–28.3°
α = 73.674 (1)°	µ = 1.03 mm^−^^1^
β = 79.066 (1)°	*T* = 293 K
γ = 68.634 (1)°	Block, orange
*V* = 1442.67 (9) Å^3^	0.30 × 0.23 × 0.11 mm

### Data collection

**Table:**

Bruker SMART APEX CCD diffractometer	6610 independent reflections
Radiation source: fine-focus sealed tube	5863 reflections with *I* > 2σ(*I*)
Graphite monochromator	*R*_int_ = 0.022
ω scan	θ_max_ = 27.5°, θ_min_ = 1.3°
Absorption correction: multi-scan (*SADABS*; Bruker, 2000)	*h* = −10→10
*T*_min_ = 0.747, *T*_max_ = 0.895	*k* = −14→14
19016 measured reflections	*l* = −21→21

### Refinement

**Table:**

Refinement on *F*^2^	Primary atom site location: structure-invariant direct methods
Least-squares matrix: full	Secondary atom site location: difference Fourier map
*R*[*F*^2^ > 2σ(*F*^2^)] = 0.027	Hydrogen site location: inferred from neighbouring sites
*wR*(*F*^2^) = 0.069	H-atom parameters constrained
*S* = 1.03	*w* = 1/[σ^2^(*F*_o_^2^) + (0.0363*P*)^2^ + 0.3766*P*] where *P* = (*F*_o_^2^ + 2*F*_c_^2^)/3
6610 reflections	(Δ/σ)_max_ = 0.003
326 parameters	Δρ_max_ = 0.50 e Å^−^^3^
3 restraints	Δρ_min_ = −0.32 e Å^−^^3^

### Special details

**Table:**

Geometry. All e.s.d.'s (except the e.s.d. in the dihedral angle between two l.s. planes) are estimated using the full covariance matrix. The cell e.s.d.'s are taken into account individually in the estimation of e.s.d.'s in distances, angles and torsion angles; correlations between e.s.d.'s in cell parameters are only used when they are defined by crystal symmetry. An approximate (isotropic) treatment of cell e.s.d.'s is used for estimating e.s.d.'s involving l.s. planes.
Refinement. Refinement of *F*^2^ against ALL reflections. The weighted *R*-factor *wR* and goodness of fit *S* are based on *F*^2^, conventional *R*-factors *R* are based on *F*, with *F* set to zero for negative *F*^2^. The threshold expression of *F*^2^ > σ(*F*^2^) is used only for calculating *R*-factors(gt) *etc*. and is not relevant to the choice of reflections for refinement. *R*-factors based on *F*^2^ are statistically about twice as large as those based on *F*, and *R*- factors based on ALL data will be even larger.

### Fractional atomic coordinates and isotropic or equivalent isotropic displacement parameters (Å^2^)

**Table:**

	*x*	*y*	*z*	*U*_iso_*/*U*_eq_	Occ. (<1)
Pd1	0.505146 (17)	0.668916 (13)	0.771823 (8)	0.02941 (5)	
Cl1	0.25945 (8)	0.81046 (6)	0.82912 (5)	0.06129 (17)	
Cl2	0.34922 (7)	0.55828 (5)	0.73749 (4)	0.04605 (13)	
P1	0.74985 (6)	0.53495 (5)	0.72429 (3)	0.03260 (11)	
P2	0.67020 (6)	0.76776 (5)	0.79571 (3)	0.03221 (11)	
C1	0.7524 (3)	0.5243 (2)	0.61872 (13)	0.0396 (5)	
C2	0.7793 (3)	0.6212 (3)	0.55295 (15)	0.0558 (6)	
H2	0.8028	0.6887	0.5630	0.067*	
C3	0.7714 (4)	0.6179 (3)	0.47218 (17)	0.0725 (8)	
H3	0.7894	0.6834	0.4281	0.087*	
C4	0.7375 (3)	0.5199 (4)	0.45682 (18)	0.0749 (9)	
H4	0.7309	0.5189	0.4024	0.090*	
C5	0.7133 (4)	0.4230 (3)	0.5210 (2)	0.0759 (9)	
H5	0.6921	0.3552	0.5099	0.091*	
C6	0.7196 (3)	0.4237 (3)	0.60288 (17)	0.0587 (6)	
H6	0.7020	0.3575	0.6465	0.070*	
C7	0.8124 (3)	0.37111 (19)	0.78653 (14)	0.0411 (5)	
C8	0.9678 (4)	0.2825 (2)	0.76568 (18)	0.0645 (7)	
H8	1.0381	0.3068	0.7189	0.077*	
C9	1.0188 (4)	0.1582 (3)	0.8140 (2)	0.0783 (9)	
H9	1.1229	0.0991	0.7995	0.094*	
C10	0.9171 (5)	0.1225 (3)	0.8827 (2)	0.0771 (9)	
H10	0.9504	0.0383	0.9143	0.093*	
C11	0.7664 (4)	0.2098 (3)	0.9054 (2)	0.0791 (9)	
H11	0.6995	0.1858	0.9537	0.095*	
C12	0.7125 (3)	0.3341 (2)	0.85681 (17)	0.0583 (6)	
H12	0.6083	0.3925	0.8719	0.070*	
C13	0.7402 (3)	0.7045 (2)	0.89926 (12)	0.0377 (4)	
C14	0.8598 (3)	0.7441 (3)	0.92091 (17)	0.0590 (6)	
H14	0.9046	0.8027	0.8821	0.071*	
C15	0.9125 (4)	0.6965 (4)	1.0000 (2)	0.0791 (10)	
H15	0.9929	0.7230	1.0147	0.095*	
C16	0.8465 (4)	0.6107 (3)	1.05672 (18)	0.0801 (10)	
H16	0.8803	0.5805	1.1104	0.096*	
C17	0.7318 (4)	0.5686 (3)	1.03572 (16)	0.0731 (9)	
H17	0.6912	0.5073	1.0743	0.088*	
C18	0.6754 (3)	0.6170 (2)	0.95701 (14)	0.0533 (6)	
H18	0.5942	0.5905	0.9432	0.064*	
C19	0.5851 (3)	0.9401 (2)	0.78165 (15)	0.0441 (5)	
C20	0.5855 (4)	1.0192 (2)	0.70303 (19)	0.0670 (7)	
H20	0.6291	0.9842	0.6566	0.080*	
C21	0.5198 (5)	1.1521 (3)	0.6938 (3)	0.0915 (11)	
H21	0.5215	1.2056	0.6408	0.110*	
C22	0.4539 (5)	1.2048 (3)	0.7599 (3)	0.0980 (13)	
H22	0.4109	1.2938	0.7524	0.118*	
C23	0.4503 (5)	1.1276 (3)	0.8380 (3)	0.0906 (11)	
H23	0.4042	1.1641	0.8836	0.109*	
C24	0.5150 (4)	0.9944 (3)	0.8497 (2)	0.0661 (7)	
H24	0.5113	0.9420	0.9030	0.079*	
C25	0.9282 (3)	0.5915 (2)	0.72475 (14)	0.0420 (5)	
H25A	0.9877	0.5425	0.7739	0.050*	
H25B	1.0091	0.5765	0.6762	0.050*	
C26	0.8655 (3)	0.7338 (2)	0.72412 (13)	0.0398 (5)	
H26A	0.8417	0.7850	0.6683	0.048*	
H26B	0.9529	0.7552	0.7416	0.048*	
O1	0.8108 (4)	0.9128 (2)	0.52539 (14)	0.1089 (9)	
S1	0.76772 (16)	0.97151 (11)	0.43853 (6)	0.0890 (3)	0.8976 (18)
C27	0.9635 (7)	0.9661 (6)	0.3762 (3)	0.1181 (18)	0.8976 (18)
H27A	1.0259	0.8793	0.3716	0.177*	0.8976 (18)
H27B	1.0304	0.9957	0.4015	0.177*	0.8976 (18)
H27C	0.9405	1.0204	0.3218	0.177*	0.8976 (18)
C28	0.6941 (10)	1.1385 (4)	0.4309 (3)	0.173 (3)	0.8976 (18)
H28A	0.5839	1.1627	0.4621	0.259*	0.8976 (18)
H28B	0.6844	1.1833	0.3735	0.259*	0.8976 (18)
H28C	0.7739	1.1605	0.4529	0.259*	0.8976 (18)
S1'	0.8125 (14)	1.0323 (6)	0.4620 (5)	0.0890 (3)	0.1024 (18)
C27'	0.880 (7)	0.957 (6)	0.378 (3)	0.1181 (18)	0.1024 (18)
H27D	1.0007	0.9093	0.3777	0.177*	0.1024 (18)
H27E	0.8594	1.0212	0.3264	0.177*	0.1024 (18)
H27F	0.8176	0.8996	0.3830	0.177*	0.1024 (18)
C28'	0.622 (4)	1.075 (5)	0.416 (3)	0.173 (3)	0.1024 (18)
H28D	0.5292	1.1302	0.4454	0.259*	0.1024 (18)
H28E	0.5966	0.9987	0.4188	0.259*	0.1024 (18)
H28F	0.6355	1.1193	0.3589	0.259*	0.1024 (18)

### Atomic displacement parameters (Å^2^)

**Table:**

	*U*^11^	*U*^22^	*U*^33^	*U*^12^	*U*^13^	*U*^23^
Pd1	0.02598 (8)	0.02756 (8)	0.03349 (9)	−0.00798 (6)	−0.00470 (5)	−0.00518 (6)
Cl1	0.0388 (3)	0.0489 (3)	0.0917 (5)	−0.0079 (2)	0.0116 (3)	−0.0302 (3)
Cl2	0.0400 (3)	0.0497 (3)	0.0574 (3)	−0.0209 (2)	−0.0106 (2)	−0.0140 (2)
P1	0.0292 (2)	0.0319 (2)	0.0360 (3)	−0.0073 (2)	−0.00501 (19)	−0.0095 (2)
P2	0.0341 (3)	0.0296 (2)	0.0341 (3)	−0.0124 (2)	−0.0048 (2)	−0.0057 (2)
C1	0.0316 (10)	0.0470 (12)	0.0391 (11)	−0.0070 (9)	−0.0028 (8)	−0.0168 (9)
C2	0.0657 (16)	0.0572 (15)	0.0435 (13)	−0.0190 (12)	−0.0054 (11)	−0.0119 (11)
C3	0.076 (2)	0.094 (2)	0.0397 (14)	−0.0225 (17)	−0.0052 (13)	−0.0122 (14)
C4	0.0483 (15)	0.125 (3)	0.0518 (16)	−0.0118 (16)	−0.0026 (12)	−0.0455 (18)
C5	0.0706 (19)	0.100 (2)	0.081 (2)	−0.0303 (18)	−0.0044 (16)	−0.056 (2)
C6	0.0609 (16)	0.0675 (17)	0.0606 (15)	−0.0280 (13)	−0.0019 (12)	−0.0280 (13)
C7	0.0420 (11)	0.0322 (10)	0.0478 (12)	−0.0070 (9)	−0.0131 (9)	−0.0085 (9)
C8	0.0594 (16)	0.0461 (14)	0.0703 (17)	0.0026 (12)	−0.0053 (13)	−0.0137 (13)
C9	0.078 (2)	0.0410 (15)	0.101 (2)	0.0086 (14)	−0.0300 (18)	−0.0182 (15)
C10	0.094 (2)	0.0361 (13)	0.100 (2)	−0.0154 (15)	−0.052 (2)	0.0062 (15)
C11	0.084 (2)	0.0604 (18)	0.081 (2)	−0.0317 (17)	−0.0205 (17)	0.0196 (16)
C12	0.0531 (15)	0.0455 (13)	0.0656 (16)	−0.0131 (11)	−0.0091 (12)	0.0014 (12)
C13	0.0366 (11)	0.0408 (11)	0.0351 (10)	−0.0087 (9)	−0.0056 (8)	−0.0117 (8)
C14	0.0565 (15)	0.0703 (17)	0.0601 (15)	−0.0226 (13)	−0.0163 (12)	−0.0210 (13)
C15	0.0661 (19)	0.104 (3)	0.076 (2)	−0.0095 (18)	−0.0327 (16)	−0.043 (2)
C16	0.071 (2)	0.102 (3)	0.0429 (15)	0.0130 (18)	−0.0222 (14)	−0.0224 (16)
C17	0.0649 (18)	0.086 (2)	0.0373 (13)	−0.0029 (15)	−0.0014 (12)	0.0021 (13)
C18	0.0480 (13)	0.0631 (15)	0.0407 (12)	−0.0158 (12)	−0.0032 (10)	−0.0036 (11)
C19	0.0403 (11)	0.0307 (10)	0.0646 (14)	−0.0139 (9)	−0.0121 (10)	−0.0085 (10)
C20	0.0733 (18)	0.0414 (13)	0.0762 (19)	−0.0169 (13)	−0.0125 (15)	0.0028 (13)
C21	0.089 (2)	0.0416 (16)	0.124 (3)	−0.0207 (16)	−0.023 (2)	0.0172 (18)
C22	0.075 (2)	0.0347 (15)	0.183 (4)	−0.0124 (15)	−0.031 (3)	−0.019 (2)
C23	0.084 (2)	0.0547 (18)	0.140 (3)	−0.0040 (16)	−0.019 (2)	−0.054 (2)
C24	0.0710 (18)	0.0453 (14)	0.0830 (19)	−0.0094 (13)	−0.0119 (15)	−0.0267 (14)
C25	0.0282 (10)	0.0505 (12)	0.0492 (12)	−0.0116 (9)	−0.0042 (9)	−0.0158 (10)
C26	0.0379 (11)	0.0453 (12)	0.0392 (11)	−0.0205 (9)	−0.0014 (8)	−0.0070 (9)
O1	0.171 (3)	0.0846 (16)	0.0628 (14)	−0.0520 (18)	−0.0197 (15)	0.0146 (12)
S1	0.1244 (9)	0.0899 (7)	0.0686 (6)	−0.0583 (7)	−0.0235 (6)	−0.0026 (5)
C27	0.119 (4)	0.164 (5)	0.083 (3)	−0.062 (4)	0.002 (3)	−0.033 (3)
C28	0.277 (8)	0.071 (3)	0.097 (4)	0.021 (4)	−0.036 (4)	0.000 (3)
S1'	0.1244 (9)	0.0899 (7)	0.0686 (6)	−0.0583 (7)	−0.0235 (6)	−0.0026 (5)
C27'	0.119 (4)	0.164 (5)	0.083 (3)	−0.062 (4)	0.002 (3)	−0.033 (3)
C28'	0.277 (8)	0.071 (3)	0.097 (4)	0.021 (4)	−0.036 (4)	0.000 (3)

### Geometric parameters (Å, º)

**Table:**

Pd1—P2	2.2336 (5)	C16—H16	0.9300
Pd1—P1	2.2355 (5)	C17—C18	1.384 (3)
Pd1—Cl1	2.3481 (6)	C17—H17	0.9300
Pd1—Cl2	2.3613 (5)	C18—H18	0.9300
P1—C1	1.808 (2)	C19—C20	1.377 (3)
P1—C7	1.813 (2)	C19—C24	1.387 (4)
P1—C25	1.840 (2)	C20—C21	1.394 (4)
P2—C19	1.802 (2)	C20—H20	0.9300
P2—C13	1.808 (2)	C21—C22	1.344 (5)
P2—C26	1.829 (2)	C21—H21	0.9300
C1—C2	1.381 (3)	C22—C23	1.364 (5)
C1—C6	1.382 (3)	C22—H22	0.9300
C2—C3	1.384 (4)	C23—C24	1.391 (4)
C2—H2	0.9300	C23—H23	0.9300
C3—C4	1.355 (5)	C24—H24	0.9300
C3—H3	0.9300	C25—C26	1.520 (3)
C4—C5	1.360 (5)	C25—H25A	0.9700
C4—H4	0.9300	C25—H25B	0.9700
C5—C6	1.390 (4)	C26—H26A	0.9700
C5—H5	0.9300	C26—H26B	0.9700
C6—H6	0.9300	O1—S1	1.479 (2)
C7—C12	1.373 (3)	O1—S1'	1.484 (2)
C7—C8	1.386 (3)	S1—C28	1.760 (5)
C8—C9	1.383 (4)	S1—C27	1.766 (5)
C8—H8	0.9300	C27—H27A	0.9600
C9—C10	1.359 (5)	C27—H27B	0.9600
C9—H9	0.9300	C27—H27C	0.9600
C10—C11	1.365 (5)	C28—H28A	0.9600
C10—H10	0.9300	C28—H28B	0.9600
C11—C12	1.386 (4)	C28—H28C	0.9600
C11—H11	0.9300	S1'—C28'	1.759 (5)
C12—H12	0.9300	S1'—C27'	1.762 (5)
C13—C18	1.377 (3)	C27'—H27D	0.9600
C13—C14	1.384 (3)	C27'—H27E	0.9600
C14—C15	1.380 (4)	C27'—H27F	0.9600
C14—H14	0.9300	C28'—H28D	0.9600
C15—C16	1.362 (5)	C28'—H28E	0.9600
C15—H15	0.9300	C28'—H28F	0.9600
C16—C17	1.362 (5)		
			
P2—Pd1—P1	85.603 (19)	C16—C17—H17	120.0
P2—Pd1—Cl1	90.93 (2)	C18—C17—H17	120.0
P1—Pd1—Cl1	175.75 (2)	C13—C18—C17	119.7 (3)
P2—Pd1—Cl2	175.049 (19)	C13—C18—H18	120.1
P1—Pd1—Cl2	89.95 (2)	C17—C18—H18	120.1
Cl1—Pd1—Cl2	93.61 (2)	C20—C19—C24	119.3 (2)
C1—P1—C7	106.37 (10)	C20—C19—P2	120.4 (2)
C1—P1—C25	106.61 (10)	C24—C19—P2	120.34 (19)
C7—P1—C25	104.57 (10)	C19—C20—C21	119.3 (3)
C1—P1—Pd1	114.01 (7)	C19—C20—H20	120.4
C7—P1—Pd1	115.28 (8)	C21—C20—H20	120.4
C25—P1—Pd1	109.25 (7)	C22—C21—C20	121.4 (3)
C19—P2—C13	106.06 (10)	C22—C21—H21	119.3
C19—P2—C26	106.39 (10)	C20—C21—H21	119.3
C13—P2—C26	105.79 (10)	C21—C22—C23	119.9 (3)
C19—P2—Pd1	118.01 (7)	C21—C22—H22	120.1
C13—P2—Pd1	112.48 (7)	C23—C22—H22	120.1
C26—P2—Pd1	107.32 (7)	C22—C23—C24	120.4 (3)
C2—C1—C6	119.4 (2)	C22—C23—H23	119.8
C2—C1—P1	119.55 (17)	C24—C23—H23	119.8
C6—C1—P1	121.01 (18)	C19—C24—C23	119.7 (3)
C1—C2—C3	120.1 (3)	C19—C24—H24	120.1
C1—C2—H2	120.0	C23—C24—H24	120.1
C3—C2—H2	120.0	C26—C25—P1	111.70 (14)
C4—C3—C2	120.5 (3)	C26—C25—H25A	109.3
C4—C3—H3	119.7	P1—C25—H25A	109.3
C2—C3—H3	119.7	C26—C25—H25B	109.3
C3—C4—C5	120.0 (3)	P1—C25—H25B	109.3
C3—C4—H4	120.0	H25A—C25—H25B	107.9
C5—C4—H4	120.0	C25—C26—P2	108.28 (14)
C4—C5—C6	120.9 (3)	C25—C26—H26A	110.0
C4—C5—H5	119.5	P2—C26—H26A	110.0
C6—C5—H5	119.5	C25—C26—H26B	110.0
C1—C6—C5	119.2 (3)	P2—C26—H26B	110.0
C1—C6—H6	120.4	H26A—C26—H26B	108.4
C5—C6—H6	120.4	S1—O1—S1'	43.2 (4)
C12—C7—C8	118.8 (2)	O1—S1—C28	104.8 (2)
C12—C7—P1	121.18 (17)	O1—S1—C27	106.6 (2)
C8—C7—P1	119.89 (19)	C28—S1—C27	95.9 (3)
C9—C8—C7	120.3 (3)	S1—C27—H27A	109.5
C9—C8—H8	119.8	S1—C27—H27B	109.5
C7—C8—H8	119.8	H27A—C27—H27B	109.5
C10—C9—C8	120.1 (3)	S1—C27—H27C	109.5
C10—C9—H9	120.0	H27A—C27—H27C	109.5
C8—C9—H9	120.0	H27B—C27—H27C	109.5
C9—C10—C11	120.2 (3)	S1—C28—H28A	109.5
C9—C10—H10	119.9	S1—C28—H28B	109.5
C11—C10—H10	119.9	H28A—C28—H28B	109.5
C10—C11—C12	120.3 (3)	S1—C28—H28C	109.5
C10—C11—H11	119.9	H28A—C28—H28C	109.5
C12—C11—H11	119.9	H28B—C28—H28C	109.5
C7—C12—C11	120.2 (3)	O1—S1'—C28'	102.2 (18)
C7—C12—H12	119.9	O1—S1'—C27'	95 (2)
C11—C12—H12	119.9	C28'—S1'—C27'	77 (3)
C18—C13—C14	119.5 (2)	S1'—C27'—H27D	109.5
C18—C13—P2	120.55 (17)	S1'—C27'—H27E	109.5
C14—C13—P2	119.91 (18)	H27D—C27'—H27E	109.5
C15—C14—C13	120.0 (3)	S1'—C27'—H27F	109.5
C15—C14—H14	120.0	H27D—C27'—H27F	109.5
C13—C14—H14	120.0	H27E—C27'—H27F	109.5
C16—C15—C14	119.8 (3)	S1'—C28'—H28D	109.5
C16—C15—H15	120.1	S1'—C28'—H28E	109.5
C14—C15—H15	120.1	H28D—C28'—H28E	109.5
C17—C16—C15	120.8 (3)	S1'—C28'—H28F	109.5
C17—C16—H16	119.6	H28D—C28'—H28F	109.5
C15—C16—H16	119.6	H28E—C28'—H28F	109.5
C16—C17—C18	120.0 (3)		
			
P2—Pd1—P1—C1	−123.79 (8)	C8—C7—C12—C11	−0.5 (4)
Cl1—Pd1—P1—C1	−159.1 (3)	P1—C7—C12—C11	−177.2 (2)
Cl2—Pd1—P1—C1	54.04 (8)	C10—C11—C12—C7	−1.6 (5)
P2—Pd1—P1—C7	112.73 (8)	C19—P2—C13—C18	122.64 (19)
Cl1—Pd1—P1—C7	77.5 (3)	C26—P2—C13—C18	−124.61 (19)
Cl2—Pd1—P1—C7	−69.44 (8)	Pd1—P2—C13—C18	−7.8 (2)
P2—Pd1—P1—C25	−4.64 (8)	C19—P2—C13—C14	−57.4 (2)
Cl1—Pd1—P1—C25	−39.9 (3)	C26—P2—C13—C14	55.4 (2)
Cl2—Pd1—P1—C25	173.19 (8)	Pd1—P2—C13—C14	172.25 (17)
P1—Pd1—P2—C19	145.15 (9)	C18—C13—C14—C15	−0.4 (4)
Cl1—Pd1—P2—C19	−37.30 (9)	P2—C13—C14—C15	179.6 (2)
Cl2—Pd1—P2—C19	119.1 (2)	C13—C14—C15—C16	0.0 (4)
P1—Pd1—P2—C13	−90.85 (7)	C14—C15—C16—C17	1.6 (5)
Cl1—Pd1—P2—C13	86.70 (7)	C15—C16—C17—C18	−2.6 (5)
Cl2—Pd1—P2—C13	−116.9 (2)	C14—C13—C18—C17	−0.6 (4)
P1—Pd1—P2—C26	25.10 (8)	P2—C13—C18—C17	179.4 (2)
Cl1—Pd1—P2—C26	−157.35 (8)	C16—C17—C18—C13	2.2 (4)
Cl2—Pd1—P2—C26	−0.9 (2)	C13—P2—C19—C20	153.5 (2)
C7—P1—C1—C2	−153.83 (19)	C26—P2—C19—C20	41.2 (2)
C25—P1—C1—C2	−42.6 (2)	Pd1—P2—C19—C20	−79.3 (2)
Pd1—P1—C1—C2	77.99 (19)	C13—P2—C19—C24	−28.0 (2)
C7—P1—C1—C6	29.3 (2)	C26—P2—C19—C24	−140.3 (2)
C25—P1—C1—C6	140.52 (19)	Pd1—P2—C19—C24	99.2 (2)
Pd1—P1—C1—C6	−98.85 (19)	C24—C19—C20—C21	1.8 (4)
C6—C1—C2—C3	0.8 (4)	P2—C19—C20—C21	−179.7 (2)
P1—C1—C2—C3	−176.1 (2)	C19—C20—C21—C22	−0.9 (5)
C1—C2—C3—C4	−0.1 (4)	C20—C21—C22—C23	−0.1 (6)
C2—C3—C4—C5	−0.8 (5)	C21—C22—C23—C24	0.3 (6)
C3—C4—C5—C6	1.2 (5)	C20—C19—C24—C23	−1.7 (4)
C2—C1—C6—C5	−0.4 (4)	P2—C19—C24—C23	179.8 (2)
P1—C1—C6—C5	176.4 (2)	C22—C23—C24—C19	0.6 (5)
C4—C5—C6—C1	−0.5 (4)	C1—P1—C25—C26	101.86 (16)
C1—P1—C7—C12	−126.3 (2)	C7—P1—C25—C26	−145.72 (15)
C25—P1—C7—C12	121.1 (2)	Pd1—P1—C25—C26	−21.79 (17)
Pd1—P1—C7—C12	1.1 (2)	P1—C25—C26—P2	42.01 (18)
C1—P1—C7—C8	56.9 (2)	C19—P2—C26—C25	−172.09 (14)
C25—P1—C7—C8	−55.7 (2)	C13—P2—C26—C25	75.39 (16)
Pd1—P1—C7—C8	−175.66 (18)	Pd1—P2—C26—C25	−44.90 (15)
C12—C7—C8—C9	1.4 (4)	S1'—O1—S1—C28	−31.7 (7)
P1—C7—C8—C9	178.3 (2)	S1'—O1—S1—C27	69.2 (7)
C7—C8—C9—C10	−0.4 (5)	S1—O1—S1'—C28'	40.1 (19)
C8—C9—C10—C11	−1.7 (5)	S1—O1—S1'—C27'	−37.4 (18)
C9—C10—C11—C12	2.6 (5)		

### Hydrogen-bond geometry (Å, º)

**Table:**

*D*—H···*A*	*D*—H	H···*A*	*D*···*A*	*D*—H···*A*
C2—H2···O1	0.93	2.49	3.348 (4)	153
C20—H20···O1	0.93	2.59	3.495 (4)	165
C26—H26*A*···O1	0.97	2.44	3.410 (3)	174
